# Continuous improvement of quality of care in pediatric diabetes: the ISPED CARD clinical registry

**DOI:** 10.1007/s00592-023-02233-6

**Published:** 2024-02-08

**Authors:** Antonio Nicolucci, Giusi Graziano, Fortunato Lombardo, Ivana Rabbone, Maria Chiara Rossi, Giacomo Vespasiani, Stefano Zucchini, Riccardo Bonfanti, The ISPED CARD Study Group, G. P. Bracciolini, V. Cherubini, A. Bobbio, S. Zucchini, T. Suprani, V. Donno, F. Lombardo, R. Bonfanti, A. Franzese, I. Rabbone, V. Graziani, M. Zampolli, I. Rutigliano, L. deSanctis, L. P. Guerraggio, R. Franceschi, G. Tornese, F. F ranco, C. Maffeis, C. Arnaldi

**Affiliations:** 1 CORESEARCH–Center for Outcomes Research and Clinical Epidemiology, Pescara, Italy; 2 Department of Human Pathology in Adult and Developmental Age “Gaetano Barresi”, University of Messina, Messina, Italy; 3 Division of Pediatrics, Department of Health Sciences, Università del Piemonte Orientale, Novara, Italy; 4 Meteda, San Benedetto del Tronto (AP), Italy; 5 Department of Pediatric, IRCCS AOU Bologna, Bologna, Italy; 6 Pediatric Diabetology Unit, Department of Pediatrics, Diabetes Research Institute, IRCCS Ospedale San Raffaele, University Vita Salute San Raffaele, Milan, Italy

**Keywords:** Type 1 diabetes, Pediatric diabetes, Quality of care, Indicators, Real-world evidence, 2BI second-generation basal insulins

## Abstract

**Aim:**

In Italy, the ISPED CARD initiative was launched to measure and improve quality of care in children and adolescents with type 1 diabetes.

**Methods:**

Process and outcome indicators and the related information derived from electronic medical records were identified. A network of pediatric diabetes centers was created on a voluntary basis.

**Results:**

Overall, 20 centers provided data on 3284 patients aged <  = 18 years. HbA1c was monitored ≥ 2/year in 81.2% of the cases. BMI was monitored ≥ 1/year in 99.0%, lipid profile in 45.3%, and blood pressure in 91.7%. Pubertal status, albuminuria, eye examination, and screening of celiac disease and thyroiditis were underreported. From 2017 to 2021, average HbA1c levels decreased from 7.8 ± 1.2 to 7.6 ± 1.3%, while patients with LDL cholesterol > 100 mg/dl increased from 18.9 to 36.7%. Prevalence of patients with elevated blood pressure and BMI/SDS values also increased. In 2021, 44.7% of patients were treated with the newest basal insulins, while use of regular human insulin had dropped to 7.7%. Use of insulin pump remained stable (37.9%).

**Conclusions:**

This report documents the feasibility of the ISPED CARD initiative and shows lights and shadows in the care provided. Improving care, increasing number of centers, and ameliorating data recording represent future challenges.

**Supplementary Information:**

The online version contains supplementary material available at 10.1007/s00592-023-02233-6.

## Introduction

Diabetes is one of the most frequent chronic diseases in childhood and adolescence. In Italy, the most common form in childhood is represented by type 1 diabetes (93%), followed by monogenic diabetes (6%), while type 2 diabetes accounts for less than 1% of the cases [1].

In 1997, the RIDI (Italian Registry on Insulin-Dependent Diabetes) was established with the purpose of collecting epidemiological data on new cases of diabetes mellitus in the 0–14 age group [2]. Data from the RIDI show a progressive increase in the incidence of type 1 diabetes, exceeding 3.6% per year in the period 1990–1999 [3] and an increase of about 3% per year in the period 1990–2003 [4]. The prevalence in children aged 0–18 years is between one case per 1000 individuals in peninsular Italy and 3–4 per 1000 individuals in Sardinia [5].

Caring for a child suffering from diabetes is demanding, since it involves the whole family nucleus and requires the presence of a group of specialists (Diabetes Team) taking charge of all the aspects (technical, psychological, nutritional, medical, etc.) that contribute to the care of the child with diabetes [6].

Despite the need for different professional figures within the diabetes team, the effective availability of all the skills is limited to a few large, highly specialized structures (Regional Reference Centers).

On the other hand, a large body of evidence documents the importance of good metabolic control and cardiovascular risk factors control, maintained over time, to prevent and/or slow down chronic complications, which represent the main cause of morbidity, mortality and resource utilization for people with diabetes [7].

Several scientific societies and associations involved in diabetes care produce recommendations for clinical practice based on scientific evidence [8–10], in order to provide an important reference tool for defining care pathways and ensuring clinical effectiveness conjugated with a correct use of the available resources. However, the mere dissemination of guidelines and recommendations may not be sufficient to influence and optimize clinical practice [11]. In fact, there may be many potential factors affecting quality of the care provided, such as the fragmentation of care pathways, insufficient economic and human resources, or specific characteristics of the patients. As a result, a large proportion of people with diabetes show suboptimal levels of metabolic control and major cardiovascular risk factors [12–14].

Given these premises, and in the light of the increasing use of information technology resources in health care, there is a need to integrate tools for continuous monitoring of the quality of care into routine clinical practice. The measurement of the gap between the ideal quality of care, represented by the recommended targets, and the quality of care provided, together with an in-depth analysis of the possible causes of this gap, can represent a powerful tool for inducing effective changes in clinical practice.

Several international public and private health organizations have promoted initiatives to measure and improve the quality of care in people with diabetes; they are based on the use of “Quality Indicators,” i.e., a series of parameters from which it is possible to establish the “dimensions of the quality of care” [15–19].

In the pediatric age, large patient databases are increasingly being developed. In Europe, the prospective diabetes follow-up registry (DPV) was developed in Germany and Austria [20]. The SWEET project (Better Control in Pediatric and Adolescent DiabeteS: Working to CrEate CEnTers of Reference) was established in 2008 as a 3-year EU public health program, with the purpose of harmonizing care of children with T1D through establishing “centers of reference” in European countries [21]. Subsequently, the project has spread out across the world including centers in Asia, North America, Africa, and Australia. In USA, the T1D Exchange Quality Improvement Collaborative (T1DX-QI) involves nearly 50 pediatric and adult endocrinology centers across the country [22].

In Italy, the Association of Diabetologists (AMD) has moved in this direction in the field of adult diabetes, with the aim of spreading not only the tools, but also and above all the culture of the regular measurement of these indicators to promote the monitoring and continuous improvement of care through the creation of the AMD ANNALS initiative [13–15].

The Italian Society of Pediatric Endocrinology and Diabetology (SIEDP), and in particular the study group of pediatric diabetology, launched a new initiative (Italian Society of Pediatric Endocrinology Diabetology Continuous clinicAl monitoRing of Diabetes, ISPED CARD) with the objective to promote the monitoring and continuous improvement of quality of care for children and adolescents with diabetes mellitus.

Aim of this paper is to describe the Italian initiative, the baseline characteristics of children and adolescents with type 1 diabetes (T1D) and quality of care indicators.

## Methods

### The Italian healthcare system

All Italian citizens are covered by a government health insurance and are registered with a general practitioner (GP). Primary care for diabetes is provided by pediatric diabetes outpatient clinics (PDOCs). Within the Italian healthcare system, about 60 PDOCs are in operation.

### The ISPED CARD initiative

The initiative envisaged three steps: the creation of a standard set of indicators, the definition of the minimum dataset, and the creation of the network of centers.

#### Identification of ISPED CARD indicators

The first step of the initiative consisted in the identification of an appropriate set of indicators, characterized by their ability to describe relevant aspects of diabetes care and the possibility of being measured in a valid, standardized, accurate, and reproducible way [15, 18].

Furthermore, the indicators were selected on the basis of the level of evidence linking them to a corresponding relevant clinical outcome [16].

Various types of measures have been identified and defined according to the type of information they allow to detect: alongside general descriptive indicators of the population under study, process and outcome indicators have been identified. Process measures include the diagnostic, preventive, therapeutic and rehabilitative procedures performed. Outcome measures are defined as those parameters that allow for the evaluation of favorable or adverse changes in the real or potential state of health of a person, group or community, which can be attributed to the healthcare received. The outcome measures can in turn be divided into intermediate measures (i.e., short term, for example metabolic control, blood pressure values, cholesterol values) and final measures (such as microvascular complications and ketoacidosis). Complete list of indicators selected on the basis of the principles described above is shown in supplementary Table 1.

#### Production of the ISPED CARD data file

Along with the list of indicators, the “standard set” of information on diabetes, risk factors, complications and therapies has been defined. The data set includes information collected during normal clinical practice, necessary for the construction of each quality indicator.

A specific list of data called “ISPED CARD data file” was therefore defined. In this way, a wide range of clinical data is extracted from electronic medical records through a specifically developed software in an automatic, standardized and strictly anonymous way. Basically, the ISPED CARD data file is able to produce a set of information whose format and/or unit of measure is exactly defined; the system utilizes available universal codings, such as the ICD-9-CM and ATC codes to unambiguously express pathologies and drug classes, in order to establish efficient comparisons between different structures and/or between different healthcare contexts.

The extraction software produces a file sent by clinicians to ISPED CARD every 2 years through a dedicated portal. Each center is able to access with personal credentials consisting of a center code and password, to protect privacy. The data uploaded to the portal are encrypted, and the portal is managed according to the most up-to-date guarantees of security and data protection.

#### Creation of the network of diabetes centers

A network of pediatric diabetes centers motivated to join the initiative was created on a voluntary basis and without any financial incentive; the only criterion for inclusion was represented by the use of a computerized medical record system capable of extracting the ISPED CARD data. The use of computerized clinical records for the routine management of patients is considered, in fact, a fundamental requirement to simplify the periodic description of care profiles and above all to integrate it into the context of daily outpatient activity [15, 22].

Participating centers are identified only by a numerical code assigned by an ISPED CARD delegate who has no direct access to the extracted data; on the other hand, the personnel who analyzes the data is not able to trace the names of the centers, but only the numerical codes. This procedure guarantees the anonymity of the participating services.

The extracted data are analyzed centrally and publicized through a specific monograph. The volume is distributed free of charge to all participants and published on the association's website. The content is designed to facilitate comparison and improvement of performance.

The study protocol was approved by the local Ethics Committees of all participating centers. Due to the study design and the anonymous by design database, based on Italian regulations, the signature of patient informed consent was not requested.

### Statistical methods

Overall, 20 centers provided data routinely collected from 2015 to 2021.

Quality of care indicators were measured in “active” patients, i.e., patients having at least a prescription of insulin in the index year.

Process and intermediate outcome indicators were assessed in patients with T1D aged <  = 18 years, overall and stratified by age classes (0–6, 6–12, 12–18 years).

If a subject was seen several times during the index year, the most recent values available were taken into consideration.

If not reported on the medical records, LDL values were calculated using the Friedwald formula when total cholesterol, HDL and triglycerides values were determined on the same date and if the triglycerides values did not exceed 400 mg/dl.

All the drugs considered were identified according to the ATC code. Short-acting insulins included the A10AB codes; basal insulins included codes A10AE; premix insulins included codes A10AD.

The data were summarized as mean and standard deviation (continuous variables) or as percentages (categorical variables). Since, given the large sample size, even clinically irrelevant differences could emerge as statistically significant, and given the descriptive and non-inferential nature of the analyses, no statistical test was applied to compare the characteristics of patients belonging to different age groups.

## Results

Overall, data relating to 3284 eligible patients with T1D seen by 20 pediatric diabetes centers during the 2021 calendar year were evaluated, of whom 202 (6.1%) aged 6 years or less, 1421 (43.3%) aged between 6.1 and 12 years, and 1661 (50.6%) aged between 12.1 and 18 years.

### Descriptive indicators

Descriptive data of the study population, overall and by age class, are reported in Table 1. The study population had a mean age of 12.1 ± 3.7 years, with a higher prevalence of males in all age classes. Overall, 11.0% of patients seen during 2021 were newly diagnosed; among children ≤ 6 years, one in three (36.6%) had new onset T1D.

**Table 1:** Characteristics of the study population, overall and by age class

Characteristics	Total sample	Age classes		
		≤ 6 years	6.1–12 years	12.1–18 years
No	3284	202	1421	1661
Mean number of visits in 2021 (± SD)	3.1 ± 1.6	3.3 ± 2.2	3.1 ± 1.6	3.0 ± 1.6
First access to diabetes clinic (%)	1.1	3.5	1.5	0.5
Newly diagnosed (%)	11.0	36.6	13.8	5.5
Gender (%)				
Females	45.6	42.1	46.3	45.4
Males	54.4	57.9	53.7	54.6
Mean age (years) (± SD)	12.1 ± 3.7			
Insulin treatment (%)				
CSII	37.9	45.5	38.3	36.7
MDI	57.0	48.0	55.7	59.2
Schemes with premixed insulin	5.1	6.4	6.1	4.0
Type of basal insulin (%)				
Detemir	0.1	0.0	0.2	0.0
Glargine U100	55.2	77.6	60.4	48.9
Degludec	34.2	20.4	28.0	40.5
Glargine U300	10.5	2.0	11.3	10.6
Type of short-acting insulin (%)				
Regular human	7.7	9.4	9.2	6.1
Lispro	50.6	51.7	51.7	49.5
Aspart	37.2	38.9	36.9	37.3
Glulisine	4.6	0.0	2.1	7.2

Over half of the patients (57.0%) were treated with multiple daily injections of insulin (MDI), 37.9% used an insulin pump (CSII), and 5.1% were treated with schemes including premixed insulin. The use of CSII was more common among children aged ≤ 6 years (45.5%).

Overall, 44.7% of the study population not treated with CSII was prescribed a second-generation basal insulin (2BI), either Degludec U100 or Glargine U300. As for short-acting insulin, 7.7% of the patients was still treated with regular human insulin, rather than insulin analogs.

### Process indicators

As for process indicators, pubertal status was registered in about one in three children aged 12 years or less and one in four aged between 10.1 and 18 years (Table 2). In the vast majority of the patients, HbA1c was monitored at least twice a year. Monitoring of body mass index (BMI), alone or associated with the measurement of waist circumference, was performed in almost all of the cases. During the index year, lipid profile was assessed in less than 50% of patients in all age classes, while blood pressure was recorded in the vast majority of children over 6 years of age. Albuminuria was monitored in increasing proportions of patients with increasing age, ranging between 20.8 and 41.4%. Eye examination, recommended for children over 10 years of age, was recorded in 9.9% of patients aged 6.1–12 years and 14.6% of those over 12 years.

**Table 2:** Process indicators, overall and by age class

Process indicators	Total sample	Age classes		
		≤ 6 years	6.1–12 years	12.1–18 years
Evaluation of pubertal status (%)	31.3	34.7	36.0	25.6
Number of HbA1c measures (%)				
0	4.5	8.4	4.4	4.2
1	14.2	15.8	15.2	13.2
≥ 2	81.2	75.7	80.4	82.7
Evaluation of BMI or waist circumference (%)				
None	1.0	3.0	1.2	0.6
BMI only	70.0	76.2	68.5	70.5
BMI and waist circumference	29.0	20.8	30.3	28.9
Evaluation of lipid profile (%)	45.3	44.6	46.8	44.1
Evaluation of blood pressure (%)	91.7	59.4	92.0	95.5
Evaluation of albuminuria (%)	38.6	20.8	37.7	41.4
Eye examination in patients aged ≥ 11 years	13.3	–	9.9	14.6
Patients screened for celiac disease (%)	7.5	7.4	8.0	7.0
Patients screened for thyroiditis (%)	13.6	12.4	12.6	14.7

Screening of celiac disease and thyroiditis was largely underreported in all age classes.

### Intermediate outcomes indicators

Average glycated hemoglobin (HbA1c) levels were 7.6 ± 1.3%, without differences according to age classes (Table 3). One in ten patients had HbA1c levels over 9.0%, with a slightly higher proportion among adolescents (12.0%).

**Table 3:** Intermediate outcome indicators, overall and by age class

Intermediate outcome indicators	Total sample	Age classes		
		≤ 6 years	6.1–12 years	12.1–18 years
HbA1c % (mean ± ds)	7.6 ± 1.3	7.5 ± 1.2	7.5 ± 1.3	7.6 ± 1.3
Classes of HbA1c (%)				
< = 6.0	7.1	7.0	7.5	6.9
6.1–6.5	12.1	9.2	13.6	11.1
6.6–7.0	18.8	21.1	20.3	17.2
7.1–7.5	18.9	25.4	20.0	17.2
7.6–8.0	15.5	13.0	15.2	16.1
8.1–8.5	10.9	7.6	10.7	11.5
8.6–9.0	6.4	7.0	4.4	8.1
> 9.0	10.3	9.7	8.4	12.0
Patients with LDL cholesterol > 100 mg/dl	36.7	42.7	36.1	36.5
Patients with blood pressure > 140/70 mmHg	18.2	11.7	10.1	25.5
Patients with BMI/SDS > 1.5	24.2	20.4	25.4	23.7

**Fig. 1:**
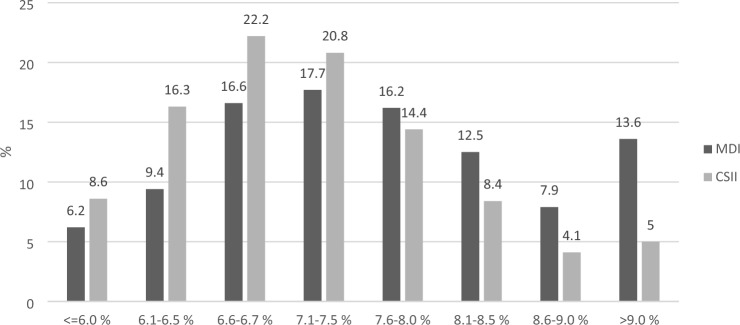
Distribution of patients by HbA1c classes and treatment modality (CSII vs. MDI)

The analysis according to the type of insulin therapy showed that average HbA1c levels were 7.3 ± 1.1% among patients treated with CSII and 7.7 ± 1.4% among those treated with MDI. The distribution of patients by HbA1c classes and treatment modality (CSII vs. MDI) is reported in Fig. 1, showing a substantially lower prevalence of elevated HbA1c levels among individuals treated with CSII. Mean HbA1c levels were lower among patients treated with CSII compared to those treated with MDI in all age classes (7.4 ± 1.4 vs. 7.7 ± 1.1% among children aged 0–6 years; 7.2 ± 1.1 vs. 7.6 ± 1.4 among those aged 6.1 to 12 years; 7.3 ± 1.1 vs. 7.8 ± 1.4% among those aged 12.1 to 18 years).

As for cardiovascular risk factors, levels of LDL cholesterol exceeded 100 mg/dl in one third of the cases overall, the proportion being even higher among children of 6 years or less (42.7%). Elevated blood pressure levels (> 140/70 mmHg) were registered in one in five patients of 12 years or less and one in four in the 12.1–18 years age range. Finally, the average standardized BMI (BMI/SDS) was 1.0 ± 0.8. Excess body weight, as indicated by a BMI/SDS value over 1.5, was documented in one in four patients over 6 years of age and one in five among younger children.

### Longitudinal trends of intermediate outcomes

The analysis of longitudinal data (time period 2017–2021) documented a transient decline in the monitoring of HbA1c during the covid pandemic in 2020 (Table 4). However, the monitoring of other parameters, such as BMI and lipid profile was not affected, and monitoring of blood pressure markedly increased over the years.

**Table 4:** Longitudinal assessment of process and intermediate outcome indicators

Indicators	Categories	2017	2018	2019	2020	2021
*N*		2228	2528	2978	3365	3284
Process indicators:						
HbA1c (%)	0 measurements	1.0	1.4	2.1	7.9	4.5
	1 measurement	8.9	7.6	9.4	26.5	14.2
	> = 2 measurements	90.0	90.9	88.5	65.6	81.2
BMI and/or waist circumference (%)	None	1.1	0.8	2.2	4.1	1.0
	BMI	75.5	85.3	77.4	80.1	70.0
	Both	23.4	13.9	20.4	15.8	29.0
Lipid profile (%)		39.6	44.6	43.8	43.3	45.3
Blood pressure (%)		52.1	54.8	66.7	77.9	91.7
CSII (%)		38.9	41.1	38.1	36.9	37.9
2BI (%)		24.8	35.2	38.7	38.3	44.7
Regular human insulin (%)		23.7	17.7	13.3	8.6	7.7
Intermediate outcomes indicators						
HbA1c % (mean ± sd)		7.8 ± 1.2	7.7 ± 1.2	7.6 ± 1.3	7.6 ± 1.3	7.6 ± 1.3
HbA1c in classes (%)	< = 6.0%	3.1	5.5	7	5.9	7.1
	6.1–6.5%	7.3	11	10.8	11.3	12.1
	6.6–7%	15.8	15.3	17.1	18.4	18.8
	7.1–7.5%	21.3	20.6	18.8	19.4	18.9
	7.6–8.0%	18.2	15.7	16.2	16.3	15.5
	8.1–8.5%	14	13.5	11.6	10.3	10.9
	8.6–9.0%	8.8	8.9	8.1	7.4	6.4
	> 9.0%	11.6	9.6	10.5	11.1	10.3
LDL-C > 100 mg/dl (%)		18.9	23.9	27.2	32.8	36.7
Blood pressure > 140/70 mmHg (%)		16.2	13.0	16.8	19.0	18.2
BMI/SDS > 1.5 (%)		19.0	20.6	21.5	23.3	24.2

As for insulin therapy, a growing proportion of patients was treated with 2BI, while the use of regular human insulin dropped from 23.7% in 2017 to 7.7% in 2021. The prevalence of use of CSII remained stable over the years. Relative to intermediate outcomes, a slight decrease in average HbA1c levels from 7.8 ± 1.2 in 2017 to 7.6 ± 1.3 in 2021 was documented (Table 4). Of note, the proportion of patients with LDL cholesterol levels over 100 mg/dl increased progressively from 18.9% in 2017 to 36.7% in 2021. The prevalence of patients with elevated blood pressure and BMI/SDS values also increased over time.

## Conclusions

Preliminary data from the ISPED CARD document the feasibility of continuous quality improvement initiative involving pediatric diabetes centers. The first data extraction involved over 3200 children and adolescents with T1D seen during 2021. According to the estimates of the International Diabetes Federation, in 2022 in Italy there were 12119 individuals with T1D below the age of 20 years [23]. Therefore, the initiative described quality of care indicators in over one-fourth of all T1D children/adolescents in the country. Considering the increasing number of participating centers, the representativeness of the study population will further increase in the years to come.

In parallel with the expansion of the number of centers involved, educational activities are ongoing to improve data quality. In fact, while 39 indicators were identified to describe and monitor diabetes care, ten indicators could not be evaluated due to the lack of information in EMRs. In particular, information on hypoglycemic and ketoacidosis episodes will deserve particular attention. Furthermore, some procedures such as for example the screening of celiac disease or thyroiditis, were registered only in a minority of cases, suggesting a substantial underreporting of this information. On the other hand, selective reporting of pathological values cannot be ruled out.

Along with an improvement in data registration, additional indicators could also be envisaged. In particular, with the widespread use of CGM and FGM, information on glucometric variables, such as the time above, below, or in range will provide additional, important insights regarding the outcomes of T1D care.

Despite these limitations, the initiative provided important insights regarding the care provided to children/adolescents with T1D. The study documented an improvement over time in average HbA1c levels, in parallel with an increasing use of more recent insulin formulations; on the other hand, the use of CSII does not seem to have increased over time. It can be speculated that improvements in metabolic control could be at least partially attributable to an increasing use of continuous glucose monitoring (CGM) and integrated systems. From this point of view, more accurate reporting of the use of technologies in EMRs represents an important aim of the initiative.

The study also provides a worrisome picture regarding cardiovascular risk factors. A non-negligible proportion of children and adolescents with T1D show elevated BMI/SDS (24.2%), blood pressure (18.2%) and LDL cholesterol levels (36.7%), suggesting the need to increase the attention to the control of cardiovascular risk factors. In this respect, young children pose additional problems, considering the limitations in the possibility of prescribing lipid-lowering agents in this age category.

In the SWEET registry, including key quality indicators relative to 22 centers from Europe, Australia, Canada, and India in youth with type 1 diabetes (T1D), an analysis was performed on 13654 persons with T1D < 25 years, 52% male, age 13.3 years, diabetes duration 4.2 years, average BMI/SDS 0.42 [24]. The study documented in 2016–2018 an adjusted mean HbA1c of 7.8% in the overall sample, with a prevalence of use of insulin pump of 41.8%. In all age classes, CSII users showed lower HbA1c levels than persons treated with MDI (7.3 vs. 7.6% in children < 6 years, 7.5 vs.7.8% in those aged > 6 to ≤ 12 years, and 7.7 vs. 8.2% in patients aged > 12 to ≤ 16 years). Data from ISPED CARD show that the level of metabolic control in children and adolescents in Italy is somehow better, with an average HbA1c of 7.6 ± 1.3%, without differences according to age classes. These results were obtained despite a slightly lower prevalence of use of CSII in patients over 6 years. In line with the results of SWEET, we documented that CSII users had better HbA1c levels in all age classes. Italian children and adolescents show markedly higher average BMI/SDS levels (1.0) compared to the SWEET population (0.42).

In USA, in the context of the T1D exchange registry, data from 2017 to 2022 relative to 16 pediatric clinics and 25383 children and adolescents (up to 18 years of age) with a type 1 diabetes duration < 1 year were analyzed (mean age 13.3 years, mean diabetes duration 8 years) [25]. Overall, 18% of the patients had HbA1c < 7%, 44% had HbA1c levels between 7 and 9%, and 38% had HbA1c levels over 9%. The prevalence of persons with HbA1c > 9.0% reached 42.2% in the age range 13–18 years. Prevalence of use of CSII was 40% among patients with HbA1c < 7%, 47% among those with HbA1c between 7 and 9%, and 27% among those with HbA1c > 9.0%.

Compared with data from this comprehensive registry, we documented that in Italy, the proportion of children and adolescents with very poor metabolic control (HbA1c > 9.0%) was markedly lower (9.7, 8.4 and 12.0% in patients aged ≤ 6 years, between 6.1 and 12 years, and between 12.1 and 18 years, respectively).

In Italy, a bill was recently issued on strategies for prevention and optimization of care and protection of children and adolescents with diabetes. The document emphasizes the need to monitor and verify quality and outcomes of diabetes care through the definition and detection of process and outcome indicators, on which to implement consequent improvement initiatives. In this respect, ISPED CARD initiative can represent an ideal source of information for policy making.

In conclusion, the ISPED CARD initiative documents lights and shadows in the care of children and adolescents with type 1 diabetes. While metabolic control appears to be satisfactory in the majority of patients, cardiovascular risk factors and the control of body weight deserve particular attention. Continuous monitoring of quality of care can represent an important tool to improve quality of care and facilitate the adoption of new technologies.

### Supplementary Information

Below is the link to the electronic supplementary material.

Supplementary file1 (DOCX 16 KB)

## Data Availability

The datasets generated during and/or analyzed during the current study are available from the corresponding author on reasonable request.

## Abbreviations

BMI: Body mass index
BMI/SDS: Standardized BMI
CSII: Continuous subcutaneous insulin infusion (insulin pump)
CGM: Continuous glucose monitoring
GP: General practitioner
HbA1c: Glycated hemoglobin
ISPED CARD: Italian society of pediatric endocrinology and diabetology continuous clinical monitoring of diabetes
LDL: Low-density lipoproteins
MDI: Multiple daily injections of insulin
PDOC: Pediatric diabetes outpatient clinics
SIEDP: Italian society of pediatric endocrinology and diabetology
