# Supplementary material for: Continuous improvement of quality of care in pediatric diabetes: the ISPED CARD clinical registry
Source: Acta Diabetol. 2024 Feb 8;61(5):599–607. doi: 10.1007/s00592-023-02233-6 (PMC11055792; doi:10.1007/s00592-023-02233-6)
Supplement: Supplementary file 1 — Supplementary file1 (DOCX 16 KB) [file 592_2023_2233_MOESM1_ESM.docx]

**Supplementary table 1**: ISPED CARD quality of care indicators

| **DESCRIPTIVE INDICATORS** |
| --- |
| 1. Distribution of patients by type of diabetes (type 1, type 2, other) |
| 1. Number of first accesses in the last 12 months |
| 1. Number of new diagnoses in the last 12 months |
| 1. Number of patients referred to the adult diabetes clinics |
| 1. Distribution by gender |
| 1. Mean age |
| 1. Distribution by age group (0-6; 6.1-12; 12.1-18; >18) |
| 1. Gene typing for non-type 1 diabetes cases (MODY 1-5; other beta cell genetic alterations) |
| 1. Proportion of patients with coeliac disease |
| 1. Proportion of patients with thyroiditis |
| 1. Proportion of patients treated with insulin pump (CSII) or multiple insulin injections (MDI) |
| 1. Proportion of patients using continuous glucose monitoring (CGM) |
| 1. Proportion of patients using flash glucose monitoring (FGM) |
| 1. Proportion of patients using only home self-monitoring of blood glucose (SMBG) |
| 1. Insulin types |
| 1. Insulin doses |
| **ACTIVITY VOLUME INDICATORS** |
| 1. Number of subjects seen in the last 12 months |
| 1. Average number of visits |
| **PROCESS INDICATORS**: Proportion of patients who during the last 12 months received: |
| 1. HLA typing for type 1 (HLADQ2 positive, DQ8 negative) |
| 1. Auxological evaluation (weight, height, BMI, pubertal stage) |
| 1. At least 2 HbA1c measurements |
| 1. Lipid profile evaluation |
| 1. Blood pressure measurement |
| 1. BMI and/or waist circumference measurement |
| 1. AER measurement (from age 11, with at least two years of disease) |
| 1. Fundus Oculi examination (from 11 years of age, with at least two years of disease) |
| 1. Coeliac disease test with antitransglutaminase |
| 1. Thyroiditis screening with TSH reflex |
| **INTERMEDIATE OUTCOME INDICATORS** (last available values in the last 12 months): |
| 1. Mean HbA1c |
| 1. Distribution of patients by 8 HbA1c classes (<=6.0, 6.1-6.5, 6.6-7.0, 7.1-7.5, 7.6-8.0, 8.1-8.5, 8.6-9.0, >9.0%) |
| 1. Proportion of patients with LDL cholesterol > 100 mg/dl |
| 1. Proportion of patients with blood pressure >140/70 mmHg |
| 1. Proportion of patients with BMI/SDS >1.5 |
| 1. Proportion of patients with AER > 20 mcg/min |
| 1. Proportion of smokers |
| **FINAL OUTCOME INDICATORS** |
| 1. Severe hypoglycemia |
| 1. Diabetic ketoacidosis (DKA) |
| 1. Proportion of patients with retinopathy |
| 1. Proportion of patients without diabetes complications |
